# A randomised, controlled, double-blind, cross-over pilot study assessing the effects of spironolactone, losartan and their combination on heart rate variability and QT dispersion in patients with chronic heart failure

**Published:** 2008-11

**Authors:** A Shehab, AA Elnour, AD Struthers

**Affiliations:** Department of Internal Medicine, Faculty of Medicine and Health Sciences, United Arab Emirates University, and Medical Institute, in affiliation with VAMED and Vienna Medical University, Abu Dhabi Health Services Company, Al Ain, United Arab Emirates; Pharmacy Department, Faculty of Medicine and Health Sciences, United Arab Emirates University, and Medical Institute, in affiliation with VAMED and Vienna Medical University, Abu Dhabi Health Services Company, Al Ain, United Arab Emirates; Department of Clinical Pharmacology and Therapeutics, Ninewells Hospital and Medical School, Dundee, Scotland

## Abstract

**Background and objective:**

The blocking of aldosterone or angiotensin II receptors improves mortality in patients with chronic heart failure. We explored whether combining losartan and spironolactone would have any added benefit on the known surrogate of mortality by using heart rate variability (HRV) and QT dispersion as our endpoints.

**Methods:**

We designed a three-phase, consecutive, randomised, controlled, double-blind, cross-over pilot study to assess the effects of losartan alone (50 mg/day), spironolactone (25 mg/day) with angiotensin converting enzyme (ACE) inhibitor and, finally, losartan with spironolactone, on HRV and QT dispersion. We enrolled eight patients (aged 47 to 72 years, mean = 63.7 years), with New York Heart Association (NYHA) class II–III heart failure and ejection fraction (EF) < 35%, in the study at a university-affiliated hospital in Dundee, Scotland. Digital 24-hour Holter recordings were analysed for time-domain HRV and the 12-lead ECG was optically scanned and digitised for analysis of QT dispersion. Evaluations were done at baseline, and at six, 12 and 18 weeks from baseline.

**Results:**

Losartan and spironolactone showed statistically significant, favourable effects on HRV, QT dispersion and mean heart rate (*p* < 0.05).

**Conclusion:**

The data showed that in these patients with heart failure, the addition of spironolactone to an ACE inhibitor, or the use of losartan on its own, or the combination of losartan plus spironolactone induced a favourable sympathovagal balance. The drugs significantly improved HRV indices and QT dispersion further, and the combination appeared to be safe. However, no significant differences were seen between the effects of each of these regimes on HRV and QT dispersion.

## Summary

Despite significant advances in the pharmacological treatment of chronic heart failure (CHF), mortality and morbidity remain high. Over the last two decades, heart failure specialists have learned that neurohormonal activation, primarily mediated through the renin-angiotensin-aldosterone system (RAAS) and the sympathetic nervous system, is a key determinant in the progression of CHF.^1^,^2^

Preliminary studies in animal models of heart failure have suggested that a more beneficial blockade of angiotensin II can be accomplished by combining the effects of an angiotensin converting enzyme (ACE) inhibitor and an angiotensin receptor blocker (ARB).^3^ These observations have led to the design of several clinical trials to ascertain whether the combination of an ARB and an ACE inhibitor might be beneficial for clinical outcomes in patients with CHF.^4^-^7^

Two studies attempted to determine whether an ARB added to an ACE inhibitor would contribute favourably to clinical outcomes in CHF.^8^,^9^ In the Valsartan Heart Failure Trial (Val-HeFT), the results indicated no significant reduction in mortality but a significant reduction in morbidity.^9^ These results seem to indicate the potential benefit of combining an ACE inhibitor with an ARB in CHF. Confirmatory trials (candesartan cilexetil in heart failure – assessment of reduction of mortality and morbidity) will help to establish this benefit. Gaudet and coworkers^10^ provided evidence for the importance of angiotensin II (losartan) and sympathetic interaction in heart rate variability responses to a stressful environment. We wanted to investigate the role of losartan (an ARB) in CHF in relation to HRV.

The activity of the RAAS is increased in most patients with CHF.^1^ A sustained increase in circulating aldosterone levels, together with dietary sodium loading is accompanied by the proliferation of fibroblasts and induction of perivascular and interstitial fibrosis^11^,^12^ This fibrosis may play a role in the reduction of systolic function, increasing ventricular stiffness, thereby impairing diastolic function and possibly generating heterogeneous intracardiac conduction defects. This has the potential for serious re-entrant arrhythmias through the inhibition of cardiac norepinephrine uptake, the augmentation of sympathetic activity, inhibition of parasympathetic traffic, and impairment of baroreceptor-mediated HRV.

ACE inhibitor-mediated reduction of aldosterone levels is weak, variable, and not sustained, regardless of whether angiotensin II levels remain suppressed.^13^ As many as 40% of patients on ACE inhibitors have persistently elevated concentrations of serum aldosterone, by way of the breakthrough generation of angiotensin II.^14^ This transient suppression and the subsequent escape phenomenon is accounted for in several ways.^15^ A major one is that ACE inhibitors increase plasma potassium, which is a well-known stimulus for aldosterone. This fibrosis can be prevented by treatment with spironolactone.^16^

Despite the belief that treatment with an aldosterone receptor blocker in conjunction with an ACE inhibitor is relatively contra-indicated because of the potential for serious hyperkalaemia, 17 the addition of an aldosterone receptor blocker (spironolactone) to standard therapy was found to be well tolerated.^18^ In the RALES trial, an aldosterone receptor blocker reduced morbidity and mortality among patients already on an ACE inhibitor, which emphasises the ineffective suppression of aldosterone production by standard doses of an ACE inhibitor. The reduction in rate of death observed in the RALES study suggests a cardioprotective effect. No significant haemodynamic differences were observed between the groups.^19^

MacFadyen and co-workers reported that spironolactone reduced the early morning increase in heart rate and collagen turnover in patients with heart failure.^20^ This suggests that spironolactone may have positive effects on HRV.

The above shows that both spironolactone and ARBs have a role to play in management of CHF. Spironolactone is definitely beneficial in severe CHF, where it improves morbidity and mortality. ARBs appear, from Val-HeFT results, to improve morbidity in NYHA class II and III patients.^9^ However, no one has compared the effects of spironolactone and ARBs on autonomic function in a longitudinal fashion.

The aim of this study was to evaluate the effect of losartan alone, of spironolactone with ACE inhibitor, and losartan with spironolactone in a three-phase, consecutive, cross-over, controlled study of patients with CHF. This was pursued by analysis of changes in their HRV and QT dispersion. Our study was designed after the ELITE I results were made public but before the ELITE II results came out.^4^,^5^ We compared the RALES approach^19^ with ELITE I/II, versus a combination of the RALES and ELITE strategies. This was the rationale behind the choice of the regimes studied here. Knowing the ELITE II and Val-Heft results now, the precise therapies investigated here would have been different, but at the time we derived this study, it was reasonable to expect that ELITE II would produce the same results as ELITE I.

## Methods

The primary objective was to assess improvement in HRV indices, QTc dispersion and mean heart rate from baseline with the addition of the study therapies to conventional treatment for CHF. We then determined any significant differences in the above effects between the studied therapies.

Patients were eligible for enrolment if they had had a history and clinical findings of heart failure for at least three months and had NYHA class III to IV heart failure, if they were clinically stable at the time of enrolment, and if their left ventricular ejection fraction was 35%. They also had to have been receiving for at least a month, a fixed-dose drug regimen for CHF that included ACE inhibitor, digoxin and furosemide. Potassium-sparing diuretics, beta-blockers and calcium channel blockers were not permitted. Beta-blockers were not routinely used in heart failure at the time of this study, and oral potassium supplements (aldosterone antagonists) were not recommended unless hypokalaemia (defined as serum potassium concentration < 3.5 mmol/l) developed.

Patients were excluded from the study if they had primary valvular heart disease, congenital heart disease, unstable angina, primary hepatic failure, type 1 diabetes mellitus, significantly abnormal clinical haematology or biochemistry results prior to starting the study, had had a myocardial infarction 30 days before the first dose of study medication, or had any life-threatening disease. Other criteria for exclusion were patients receiving regular non-steroidal anti-inflammatory drugs (NSAIDs) or aspirin (> 300 mg/day), steroids, dopamine agonists or antagonists, insulin or heparin.

The study conformed with the principles outlined in the Declaration of Helsinki, and was approved by the Tayside local research ethics committee, Dundee, Scotland. All participants gave written informed consent.

## Intervention

**Fig. 1.**
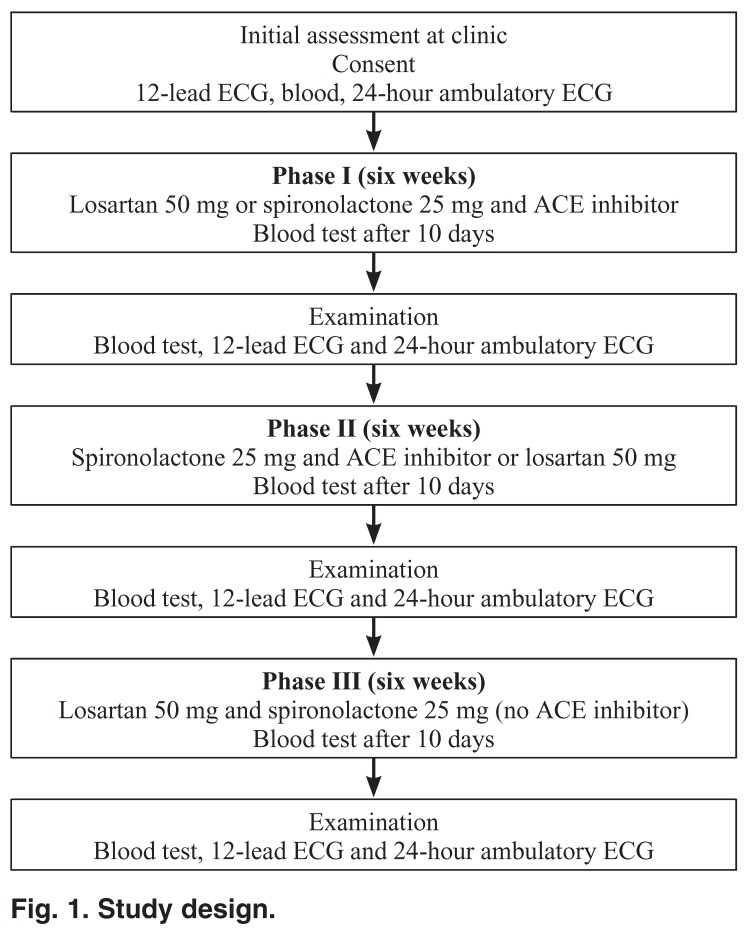
Study design.

Following this initial assessment, patients were assigned to the three-phase, consecutive, cross-over study (Fig. 1). In phase one, patients were commenced randomly on either losartan alone, 50 mg (group A), or spironolactone 25 mg plus an ACE inhibitor (group B), for six weeks. In phase two, patients were crossed over to receive the alternative, randomly allocated losartan or spironolactone for a further six weeks. In phase three (further six weeks’ duration), the combination of losartan 50 mg plus spironolactone 25 mg was administered without the ACE inhibitor.

At baseline, demographic and clinical information was obtained (Table 1). Patients were seen 10 days after starting on the therapy for each phase, to assess the urea and electrolytes for any complications (e.g. hyperkalaemia), and at the end of each phase (six-week intervals), patients were assessed clinically (quality-of-life questionnaire) and in the laboratory (blood, Holter monitoring).

**Table 1:** Baseline Characteristics Of Study Patients

*Baseline characteristics*	*Group A (n = 4) Losartan – spironolactone Mean ± 1 SD*	*Group B (n = 4) Spironolactone – losartan Mean ± 1 SD*	*p-value
Age (years)	64 ± 12	63 ± 1	NS
Women/men (%)	2/2 (50)	2/2 (50)	NS
Systolic BP (mmHg)	135 ± 19	138 ± 22	NS
Diastolic BP (mmHg)	78 ± 5	72 ± 5	NS
Pulse (beats/min)	64 ± 6	62 ± 3	NS
Serum Na (mmol/l)	137 ± 3	138 ± 1	NS
Serum K (mmol/l)	4.6 ± 0.1	4.3 ± 0.1	NS
NYHA class (%)			
II	2 (50)	4 (100)	
III	2 (50)		
LVEF (%)	28 ± 5	33 ± 4	NS
Mean heart rate	82 ± 10	68 ± 8	NS
Ventricular ectopics	346 ± 633	1793 ± 706	NS
24-h SDNN (ms)	85 ± 39	112 ± 34	NS
24-h SDANN (ms)	90 ± 47	103 ± 28	NS
24-h RMSSD (ms)	20 ± 10	25 ± 8	NS
24-h TI	20 ± 6	30 ± 8	NS
QTc dispersion	69 ± 8	70 ± 8	NS
Max QTc	436 ± 23	421 ± 6	NS
Diuretics (mg)	45 ± 25	60 ± 23	NS
ACE inhibitor (mg)	11 ± 6	8 ± 4	NS
Aspirin (mg)	94 ± 37	100 ± 43	NS
Nitrate (mg)	37 ± 17	42 ± 24	NS
Statin (mg)	17 ± 7	16 ± 5	NS

NS: not significant, BP: blood presure, LVEF: left ventricular ejection fraction, ACE: angiotensin converting enzyme, statin: HMG-Co reductase inhibitors. *p < 0.05.

## Measuring heart rate variability

At baseline and at six-week intervals thereafter, patients underwent standard Holter monitoring (Tracker^2^, Reynolds Medical Ltd, Hertford, UK). To avoid a confounding influence of cardiovascular drugs on HRV measurements, the baseline Holter recordings used for the present study were performed in all patients after 30 days of standard drug therapy. HRV analysis was performed according to established criteria from two-channel, 24-hour ambulatory electrocardiographic recordings.^21^

Tape recordings were analysed after being transferred to a computer with special software (Reynolds pathfinder 600 series workstation). After a normal QRS complex was chosen, computer-assisted rate and arrhythmia analysis was performed with an expert physician over-reading and editing. Excessive noise and artefacts were excluded, and ectopy was quantified. After analysis, the results were saved in a data file. These data files were later downloaded and analysed.

Time-domain analysis included average RR interval (normal-to-normal = NN), percentage of difference between successive normal RR intervals that were > 50 ms computed over the entire 24-hour ECG recording (pNN50), (NN50, the number of interval differences of successive NN intervals greater than 50 ms, and pNN50, the proportion derived by dividing NN50 by the total number of NN intervals), and the triangular interpolation of the NN histogram (TINN). TINN was the width of the base of graphic display of the NN histogram that was automatically drawn by the software, and was expressed in milliseconds. In addition to other time-domain indices such as the standard deviation of the average NN intervals (SDANN), the square root of the mean squared differences of successive NN intervals (RMSSD) and standard deviation in NN (SDNN) index were calculated. All of these measurements of short-term variation estimate high-frequency variations in heart rate and are therefore highly correlated.

## QT dispersion measurement

QT-interval analysis was done on 12-lead electrocardiograms (ECGs). A single observer, unaware of the diagnoses and blinded to the order of ECG recordings and other measurements, measured QT intervals in all leads. All measurements were made on 12-lead ECG rhythm strips recorded at a speed of 25 mm/sec. The QT interval was taken from the onset of the QRS to the end of the T wave (the end of the T wave was defined as the intersection of the iso-electric line and the tangent of the maximal slope on the downward limb of the T wave). If U waves were present, the QT interval was measured to the nadir of the curve between the T and U waves. The QT interval was measured in at least nine leads in three consecutive cardiac cycles.

QT intervals were corrected with Bazett’s formula (QTc = QT/RR). QTc dispersion, defined as the difference between the maximum and minimum QTc, was calculated in ECGs in which at least five leads were measurable. Adjusted QTc dispersion was measured to correct for the known dependence of the index on the number of measurable leads. The intra-observer correlations of variation of these QT indexes were under 10%.

## Statistical analysis

The calculation of sample size was based on HRV (SDNN) improvement. The number of patients required in each arm to detect with 90% power a 20% difference between the baseline and treatments was calculated to be eight.

Results were quoted as means ± SD (SEM). Normality of the distribution of the data was assessed by chi square (χ^2^) analyses with a goodness-of-fit test. Non-parametric statistical methods were used when the variables did not show a normal distribution. Changes in variables were analysed using general linear model repeated measures. If significant difference was determined overall, comparison at pre-specified time intervals was done using *post hoc* simultaneous multiple comparisons with the Bonferroni correction; also when appropriate, the paired *t*-test was used. Statistical significance was defined at the *p* < 0.05 level.

## Results

Eight patients (mean age 63.7 years; range 47–72) were admitted and completed the study. The clinical condition of the patients was evaluated by detailed history and complete physical examination. As a whole, the patients’ heart failure remained stable throughout the study. Serum potassium levels remained within the normal range throughout the study period and did not change significantly from baseline (*p* = 0.25). Table 2 illustrates that HRV indices improved significantly from baseline. There were no significant differences between these therapies with respect to their HRV indices.

**Table 2:** The Effect Of Study Medication On HRV (SDANN, TI, RMSSD) And QT Dispersion From Baseline And Between Drugs, Using Parametric And Non-Parametric Analyses

*Parameters*	*Mean difference ± SEM*	*95% CI*	p-*value*	p-*value*
SDANN				
Baseline – losartan	–27 ± 8.3	(–46, –7)	0.014	0.012
Baseline – (spiro + ACEI)	–31 ± 7.6	(–49, –13)	0.004	0.012
Baseline – (losartan + spiro)	–39 ± 9.7	(–62, –15)	0.005	0.012
Losartan – (spiro + ACEI)	–4 ± 9.7	(–27, 18)	0.65	0.4
Losartan – (losartan + spiro)	–12 ± 10.8	(–37,13)	0.32	0.6
(Spiro + ACEI) – (losartan + spiro)	–7 ± 10.4	(–32,17)	0.49	0.48
Triangular index				
Baseline – losartan	–6 ± 2.1	(–1, –1.6)	0.016	0.018
Baseline – (spiro + ACEI)	–8 ± 2.9	(–15, –1.5)	0.024	0.012
Baseline – (losartan + spiro)	–8 ± 3	(–14, –0.7)	0.034	0.043
Losartan – (spiro + ACEI)	–1.7 ± 4.1	(–11, 8)	0.68	0.77
Losartan – (losartan + spiro)	–1.1 ± 1.9	(–5, 3)	0.58	0.57
(Spiro + ACEI) – (losartan + spiro)	–0.6 ± 4.3	(–9, 10)	0.88	0.61
rMSSD				
Baseline – losartan	–2.8 ± 1.3	(–6, 0.4)	0.078	0.046
Baseline – (spiro + ACEI)	–5.7 ± 7.1	(–11, 0.2)	0.056	0.027
Baseline – (losartan + spiro)	–6 ± 1.6	(–9.8, -2.1)	0.008	0.027
Losartan – (spiro + ACEI)	–2.8 ± 2.6	(–9, 3.4)	0.31	0.29
Losartan – (losartan + spiro)	–3.1 ± 1.3	(–6, 0.04)	0.053	0.027
(Spiro + ACEI) – (losartan + spiro)	–0.25 ± 2	(–5, 4.5)	0.9	0.89
QTc dispersion				
Baseline – losartan	18 ± 3.3	(10, 27)	0.001	0.018
Baseline – (spiro + ACEI)	20 ± 3.3	(12, 29)	0.001	0.018
Baseline – (losartan + spiro)	27 ± 2.8	(20, 34)	0.0001	0.018
Losartan – (spiro + ACEI)	1.9 ± 1.3	(–1.3, 5.2)	0.2	0.23
Losartan – (losartan + spiro)	8 ± 2.7	(1.4, 14)	0.025	0.063
(Spiro + ACEI) – (losartan + spiro)	6.2 ± 2.2	(0.7, 11.6)	0.031	0.028
Ventricular ectopics				
Baseline – losartan	674 ± 326	(–98, 1447)	0.078	0.025
Baseline – (spiro + ACEI)	444 ± 362	(–413, 1302)	0.26	0.093
Baseline – (losartan + spiro)	725 ± 313	(–14, 1466)	0.053	0.017
Losartan – (spiro + ACEI)	–230 ± 179	(–655, 195)	0.24	0.4
Losartan – (losartan + spiro)	51 ± 56	(–82, 182)	0.39	0.12
(Spiro + ACEI) – (losartan + spiro)	281 ± 172	(–127, 690)	0.14	0.23

Spiro: spironolactone, ACEI: angiotensin converting enzyme inhibitor.

All measures of QT/QTc during drug therapies were closely interrelated (*r* = 0.82–0.96; all *p* < 0.025). Addition of losartan alone, spironolactone plus ACE inhibitor, and then the combination of spironolactone plus losartan to the conventional heartfailure therapy resulted in a statistically significant reduction of QT (*p* = 0.0001) and adjusted QT dispersions (QTc) (range *p* = 0.001–0.0001).

Analysis of the effects of the different therapies on QTc dispersions showed that the combination therapy (spironolactone plus losartan) produced a significant reduction in QTc dispersion, compared to losartan alone (*p* = 0.025), and also compared to spironolactone plus ACE inhibitor (*p* = 0.031). There was no significant difference in QTc dispersion between losartan alone, and spironolactone plus ACE inhibitor (*p* = 0.0.2). On the other hand, the non-adjusted QT dispersion did not reduce significantly when comparing therapies to each other (*p* = 0.064) (Table 2).

On further analysis, all the study medication except losartan had a reduction effect on QT_max_ from baseline. The combination therapy (losartan plus spironolactone) had a significant reduction effect on QT_max_ in contrast to losartan alone (*p* = 0.033).

Analysis of the effects of the trial therapies on mean heart rate and ventricular ectopics showed a significant reduction in mean heart rate from baseline: losartan alone (*p* = 0.033), spironolactone plus ACE inhibitor (*p* = 0.01), and losartan plus spironolactone (*p* = 0.025) (Table 2). Ventricular ectopics counts were not significantly changed from baseline during the study. However, the effects of the study therapies with regard to each other on mean heart rate and ventricular ectopics were not statistically significant.

It is important to note that these changes in QTc dispersion were not significantly affected by the order that losartan alone (group A) or spironolactone plus ACE inhibitor (group B) were given (*p* = 0.6).

## Discussion

This pilot study showed that, when added to standard therapy, the angiotensin II receptor antagonists and aldosterone antagonists caused favourable changes in 24-hour HRV indices, QT dispersion, mean heart rate and, to a lesser extent, ventricular ectopics. No previous studies to our knowledge investigated the trial therapies in a similar manner.

Furthermore, the combination of losartan plus spironolactone appeared safe, although it did not produce any apparent extra benefit over each drug in isolation. However, there was a favourable trend in HRV in the losartan-plus-spironolactone combination therapy and, to a lesser extent, with spironolactone plus ACE inhibitor. The combination therapies of losartan plus spironolactone had more obvious effects on QTc dispersion.

Previous studies showed a favourable outcome for spironolactone but not for losartan on autonomic indices in CHF.^22^ Recently, Val-HeFT showed that valsartan plus an ACE inhibitor reduced morbidity in CHF patients.^9^

The two groups remained clinically well throughout the period and we did not study the effect of each regime on quality of life. Subjects’ renal function (especially potassium) stayed within the normal range during the study, with no significant change from baseline in both groups. These safety data were important observations, albeit in this small number of patients.

A limitation of this study was the small sample size. Prior to recruiting, we calculated that a sample of eight patients in each arm would detect a difference of 30% with *p* = 0.05 and power at least 90%. However, we experienced difficulty in recruiting this target sample size. Despite this shortfall in recruitment, we showed significant changes from baseline.

Another limitation was that not all the treatments were randomised, and hence order effects cannot be excluded. Due to the possibility of carry-on effects, our study always had baseline at the beginning and combination therapy at the end. When deriving this study, we thought that losartan plus spironolactone might worsen renal function and therefore we decided on a step-by-step protocol, whereby the only patients who got combination therapy had already shown that they could tolerate each drug in isolation. Therefore, for safety reasons, the combination therapy was always given at the end.

As mentioned above, our study was designed mainly to see if the RALES and ELITE strategies could be combined.^4^,^5^,^19^ We wanted to see if together they produced (1) any evidence of an additional benefit over either strategy alone (as measured by HRV and QT dispersion) and (2) any additional renal and/or potassium level disorders over either strategy alone.

Unfortunately the ELITE II study gave a completely different result from the ELITE I trial and this decreased the clinical relevance of the treatments that were used in this study. Knowing the results of the ELITE II and Val-Heft trials, we would now choose to study (1) an ACE inhibitor plus spironolactone, (2) an ACE inhibitor plus valsartan and, (3) an ACE inhibitor plus valsartan plus spironolactone. However, the rectrospectorescope was not available when this study was derived and it was reasonable to expect that the ELITE II results would be similar to those of ELITE I.^4^,^5^

## Conclusions

The data derived here indicate that in patients with heart failure, the addition of spironolactone to an ACE inhibitor, the use of losartan on its own, or the combination of losartan plus spironolactone all induced a favourable sympathovagal balance. This study showed that, in addition to conventional therapy for CHF, spironolactone (ACE inhibitor) or/and losartan significantly improved HRV indices and QT dispersion further, and the combination appeared to be safe. However, no significant differences were seen between these regimes on HRV and QT dispersion.
